# Novel benzoxazole derivatives DCPAB and HPAB attenuate Th1 cell-mediated inflammation through T-bet suppression

**DOI:** 10.1038/srep42144

**Published:** 2017-02-07

**Authors:** Yeon Ji Oh, Darong Kim, Sera Oh, Eun Jung Jang, Hee Yeon Won, Hana Jeong, Mi Gyeong Jeong, Hea-Young Park Choo, Eun Sook Hwang

**Affiliations:** 1 College of Pharmacy and Graduate School of Pharmaceutical Sciences, Ewha Womans University, Seoul 03760, Korea

## Abstract

Interferon-γ (IFN-γ), a critical inflammatory cytokine, is primarily produced by T helper 1 (Th1) cells and accelerates the pathogenesis of inflammatory colitis. Pharmacological suppression of IFN-γ production attenuates dysregulated inflammatory responses and may be beneficial for treating inflammatory disease. In this study, we aimed to discover potent anti-inflammatory compounds that suppress IFN-γ production and found that the novel benzoxazole derivatives, 2-((3,4-dichlorophenyl) amino) benzo[d]xazol-5-ol (DCPAB) and 2-((3,4-hydroxyphenyl) amino) benzo[d]xazol-5-ol (HPAB), suppressed IFN-γ production by T cells. Treatment of CD4+ T cells with DCPAB and HPAB selectively inhibited Th1 cell development, and DCPAB more potently suppressed IFN-γ than HPAB did. Interestingly, DCPAB and HPAB significantly suppressed the expression of T-box containing protein expressed in T cells (T-bet) that activates IFN-γ gene transcription. DCPAB additionally suppressed transcriptional activity of T-bet on IFN-γ gene promoter, whereas HPAB had no effect on T-bet activity. IFN-γ suppressive activity of DCPAB and HPAB was impaired in the absence of T-bet but was retrieved by the restoration of T-bet in T-bet-deficient T cells. Furthermore, DCPAB and HPAB attenuated inflammatory colitis development that was induced by CD4+ T cells *in vivo*. We suggest that the novel benzoxazole derivatives, DCPAB and HPAB, may have therapeutic effects on inflammatory colitis.

Chronic inflammatory bowel diseases (IBD), such as Crohn’s disease and ulcerative colitis, have been extensively studied in attempts to identify the molecular mechanisms underlying their pathogenesis and develop effective treatments. Key features of IBD pathogenesis are a persistent and increased production of mucosal cytokines and an altered epithelial homeostasis^1^,^2^. A complex interplay of a variety of inflammatory immune and non-immune cells is involved in the disease process^3^. In particular, CD4+ T cells play a central role in the induction and progression of IBD by producing pro-inflammatory cytokines, including tumor necrosis factor (TNF)-α, interferon (IFN)-γ, and interleukin (IL)-17. CD4+ T cells are activated by stimulation with an anti-T cell receptor (TCR) antibody (Ab) and subsequently differentiated into different subsets of T helper (Th) cells in response to different inflammatory environmental cytokines. In particular, IFN-γ-producing Th1 cells are generated from CD4+ T cells upon TCR activation and are further increased by treatment with IL-12, whereas IL-17-producing Th17 cells are generated only in the presence of transforming growth factor (TGF)-β and IL-6. IFN-γ causatively induces an inflammatory response in the gut and persistently increases the production of inflammatory cytokines, including IL-17, thereby exacerbating intestinal epithelial destruction^4^,^5^,^6^,^7^.

Numerous studies have attempted to develop therapeutic Ab drugs for the suppression of inflammatory cytokines^8^, including anti-TNF-α drugs (adalimumab, certolizumab pegol, etanercept, onercept and golimumab); an anti-CD20 Ab drug (rituximab); T-cell inhibitors (abatacept); and anti-α4 integrins (natalizumab and vedolizumab). These monoclonal Ab drugs show significant efficacy in the induction of remission and maintenance of IBD, but cause adverse effects and are not tolerated well. Therefore, research into and development of novel and more effective therapies, such as small-molecule drugs, are needed for treating IBD.

Benzoxazole is an aromatic organic compound that has a benzene-fused oxazole ring structure. It is used as a starting material for the synthesis of pharmaceutical drugs with larger chemical structures. Benzoxazole derivatives have shown several biological activities, including antimicrobial, antibacterial, antiviral^9^,^10^, anti-cancer^11^,^12^,^13^,^14^, anti-tuberculosis^15^, and anti-inflammatory activity^16^. Recently, benzoxazole derivatives were reported to have the potential to control depression and neurological diseases^17^,^18^. Anti-inflammatory benzoxazole derivatives inhibited cyclooxygenase and 5-lipoxygenase activities, leading to the suppression of inflammatory responses^16^,^19^. Furthermore, benzoxazole derivatives with a nitrogen-containing heterocyclic substituent suppressed irritable bowel syndrome through the suppression of 5-HT3 receptor activity^20^. However, whether benzoxazole derivatives modulate inflammatory cytokine production and have therapeutic effects in inflammatory bowel diseases require clarification.

In the present study, we synthesized novel benzoxazole derivatives, 2-((3,4-dichlorophenyl) amino) benzo[d]xazol-5-ol (DCPAB) and 2-((3,4-hydroxyphenyl) amino) benzo[d]xazol-5-ol (HPAB), and examined their effects on inflammatory cytokine production and colitis development.

## Results

### Novel benzoxazole derivatives DCPAB and HPAB suppress IFN-γ production by splenic T cells

**Figure 1:**
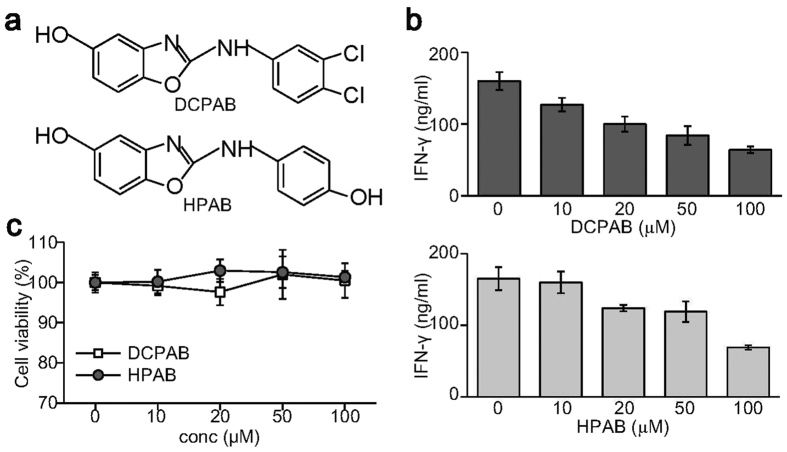
IFN-γ suppression by the novel benzoxazole derivatives DCPAB and HPAB. (**a**) Chemical structure of DCPAB and HPAB. (**b**) Spleen cells were stimulated with anti-CD3 Ab (1 μg/ml) for 24 h in the presence of either DCPAB or HPAB. Cell supernatants were used for measuring IFN-γ by ELISA. (**c**) Spleen cells were treated with DCPAB and HPAB for 24 h and subjected to cytotoxicity assay.

In an attempt to isolate potent anti-inflammatory compounds that inhibits IFN-γ production, we synthesized novel benzoxazole derivatives and purified using preparative column chromatography, followed by cell-based assay using splenic T cells. Two novel benzoxazole derivatives, DCPAB and HPAB, were isolated to suppress IFN-γ production. The structures of DCPAB and HPAB were characterized and shown in Fig. 1a. Spleen cells were treated with increasing amounts of DCPAB and HPAB after T cell receptor stimulation. Interestingly, DCPAB and HPAB suppressed IFN-γ produced by splenic T cells in a dose-dependent manner (Fig. 1b). DCPAB and HPAB had no effect on the viability of splenic T cells (Fig. 1c).

### DCPAB and HPAB inhibit IFN-γ production by CD4+ T cells

**Figure 2:**
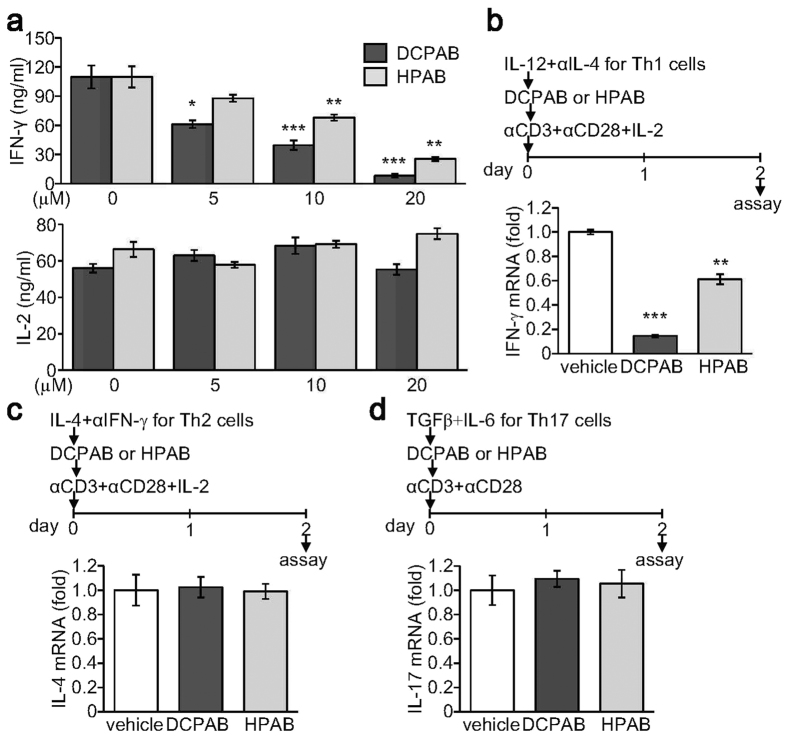
Suppression of IFN-γ production by developing Th cells by DCPAB and HPAB. CD4+ T cells were isolated from lymph nodes and spleen and stimulated with anti-CD3 (2 μg/mL) and anti-CD28 (1 μg/mL) Abs and subsequently incubated with DCPAB and HPAB for 48 h. (**a**) Cell supernatants were collected and used for measuring IFN-γ and IL-2 by ELISA. (**b–d**) CD4+ T cells were stimulated and additionally cultured under skewing conditions: IL-12 (2 ng/mL) and anti-IL-4 Ab (5 μg/mL) for Th1-skewing (**b**), IL-4 and anti- IFN-γ Ab (5 μg/mL) for Th2-skewing (**c**), and TGF-β (10 ng/mL) and IL-6 (10 ng/mL) for Th17-skewing (**d**) for 48 h. Total RNA was prepared and subjected to reverse transcription and real time-PCR to determine the relative expression level of IFN-γ (**b**), IL-4 (**c**), and IL-17 (**d**), respectively. Relative transcript level was calculated after normalization with actin level and is expressed as a value of fold-induction. *P < 0.05, **P < 0.005, ***P < 0.0005.

We then examined whether DCPAB and HPAB affected IFN-γ production by CD4+ T cells. CD4+ T cells were stimulated through a T cell receptor activation and subsequently treated with DCPAB or HPAB. IFN-γ production by activated CD4+ T cells was significantly suppressed by treatment with DCPAB or HPAB, whereas IL-2 production was not affected by both compounds (Fig. 2a). To examine the selective functions of DCPAB and HPAB, we treated CD4+ T cells with DCPAB or HPAB for 48 h under different skewing conditions. Under Th1-skewing conditions, DCPAB and HPAB significantly suppressed IFN-γ production (Fig. 2b). IFN-γ expression was suppressed by DCPAB more strongly than by HPAB (Fig. 2b). Under either Th2-skewing or Th17-skewing conditions, both DCPAB and HPAB had no effect on IL-4 and IL-17 expression (Fig. 2c,d), suggesting a specific inhibitory activity of DCPAB and HPAB on IFN-γ, not IL-4 nor IL-17, production.

### Th1 cell differentiation is inhibited by treatment with DCPAB and HPAB

**Figure 3:**
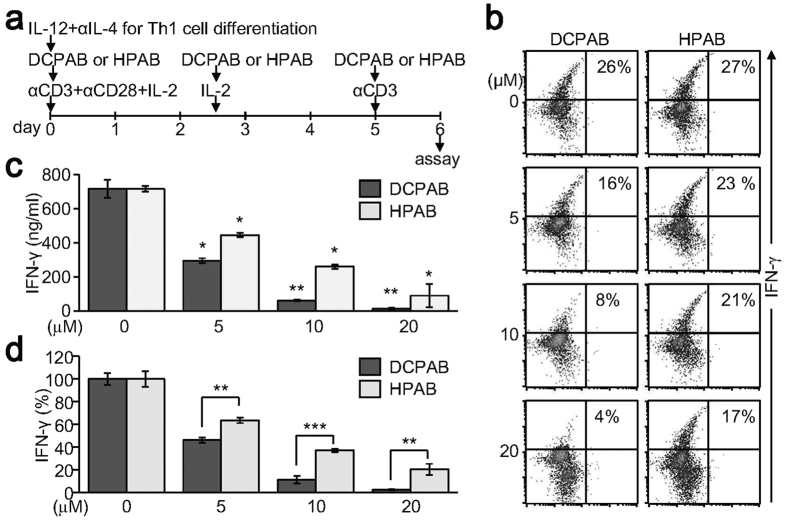
Inhibition of Th1 cell differentiation by DCPAB and HPAB. CD4+ T cells were cultured under Th1 differentiation conditions and treated with DCPAB and HPAB for 5 days. Cells were restimulated with anti-CD3 for 24 h. (**a**) Scheme for Th1 cell differentiation. (**b**) Cells were harvested and incubated with PE-conjugated anti-IFN-γ Ab, followed by flow cytometry analysis. Cell populations were quantitated using CellQuest software. (**c**) Cell supernatants were collected from the differentiated Th1 cells and used for measuring IFN-γ by ELISA. (**d**) IFN-γ production was determined in DCPAB- and HPAB-treated cells by ELISA and real time-PCR. IFN-γ suppression activity of DCPAB and HPAB was comparatively analyzed and is expressed as the percentage of suppression activity normalized to the untreated control (100%). At least three independent experiments were performed and statistically analyzed. *P < 0.05, **P < 0.005, ***P < 0.0005.

In order to confirm the specific activity of DCPAB and HPAB on IFN-γ expression, effects of DCPAB and HPAB on Th1 cell differentiation were investigated. We induced the naive CD4+ T cells to differentiate into Th1 cells and incubated the cells with DCPAB and HPAB during Th1 cell differentiation (Fig. 3a). Fully differentiated Th1 cells were determined by intracellular IFN-γ staining, followed by flow cytometry analysis. DCPAB and HPAB dose-dependently decreased IFN-γ-producing Th1 cell populations (Fig. 3b). Further analysis of the secreted IFN-γ cytokine in cell supernatants confirmed that DCPAB and HPAB significantly suppressed IFN-γ production (Fig. 3c). A quantitative and comparative analysis of DCPAB and HPAB demonstrated that DCPAB more potently suppressed IFN-γ than HPAB did (Fig. 3d).

### DCPAB suppresses both T-bet expression and activity while HPAB decreases T-bet expression

**Figure 4:**
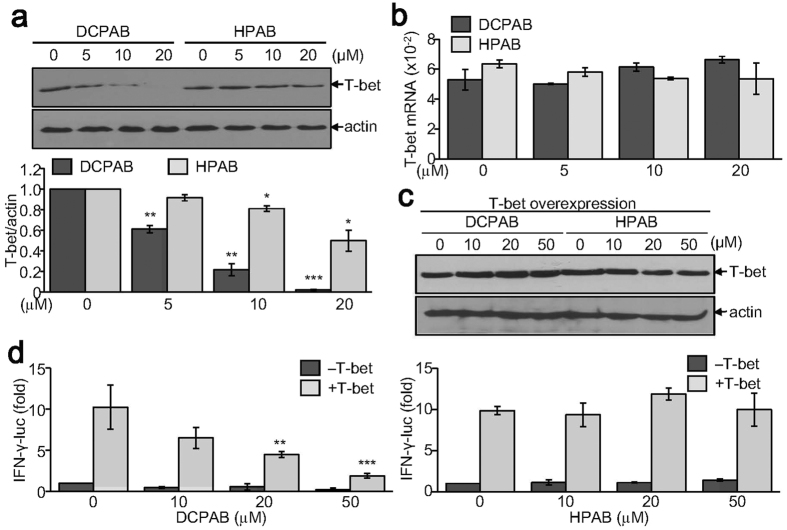
Decreased T-bet expression by treatment with DCPAB and HPAB. (**a,b**) CD4+ T cells were treated with DCPAB or HPAB for 48 h under Th1-skewing conditions. Proteins were extracted and resolved by SDS-PAGE. Protein blots were incubated with anti-T-bet Ab, followed by detection using ECL and densitometry analysis (**a**). *P < 0.05, **P < 0.005, ***P < 0.0005. Total RNA was independently prepared using TRIzol reagent and subjected to reverse transcription, followed by the quantitative analysis of T-bet mRNA (**b**). (**c,d**) HEK 293 T cells were transfected with T-bet expression vector together with INF-γ promoter-linked reporter gene (pIFN-γ-luc). The β-galactosidase expression vector, pCMVβ was also transfected for normalizing transfection efficiency. Transfected cells were incubated with DCPAB or HPAB for an additional 24 h. T-bet expression was determined by immunoblot analysis (**c**). Relative luciferase activity was calculated after normalization with β-galactosidase activity and is expressed as a fold induction from four independent experiments (**d**). **P < 0.005, ***P < 0.0005.

To investigate the molecular mechanisms underlying IFN-γ suppression and inhibition of Th1 cell differentiation by DCPAB and HPAB, we examined the effects DCPAB and HPAB on T-box containing protein expressed in T cells (T-bet) that is an essential Th1-specific transcription factor for inducing IFN-γ gene transcription^21^,^22^. We first verified that T-bet expression during Th1 cell differentiation was significantly suppressed by DCPAB and HPAB in a concentration-dependent manner (Fig. 4a). Quantitative analysis demonstrated that DCPAB potently suppressed T-bet expression. HPAB also significantly suppressed T-bet expression, but to a lesser extent (Fig. 4a). However, T-bet mRNA level was not affected by both DCPAB and HPAB (Fig. 4b), indicating the post-transcriptional regulation of T-bet by DCPAB and HPAB. We next examined whether DCPAB or HPAB affected the transcriptional activity of T-bet on IFN-γ gene promoter. Enforced T-bet expression in 394 T cells was detected by immunoblotting but its expression was not affected by treatment with DCPAB and HPAB (Fig. 4c). The IFN-γ gene promoter activity was enhanced by ectopic T-bet expression, and T-bet-induced IFN-γ promoter activity was subsequently decreased by treatment with DCPAB, but not affected by HPAB (Fig. 4d), indicating that DCPAB, not HPAB, was capable of inhibiting the transcriptional activity of T-bet.

### DCPAB and HPAB require T-bet for IFN-γ suppression

**Figure 5:**
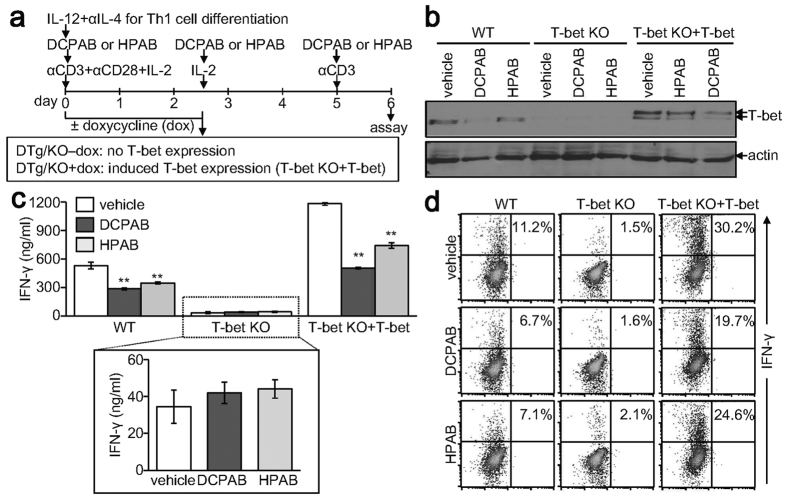
T-bet dependent IFN-γ suppression of DCPAB and HPAB. CD4+ T cells were isolated from WT, T-bet KO, and DTg/KO mice and stimulated with anti-CD3 and anti-CD28 Abs. CD4+ T cells of DTg/KO mice were additionally treated with doxycycline (dox, 1 μg/ml) for retrieving T-bet expression (T-bet KO+ T-bet). Cells were incubated with DCPAB (10 μM) or HPAB (10 μM) during Th1 cell differentiation and restimulated with anti-CD3 (1 μg/ml) according to the experimental scheme (**a**). (**b**) Protein extracts were resolved by SDS-PAGE and analyzed by immunoblotting with anti-T-bet Ab. (**c**) Cell supernatants were harvested at day 6 and used for the ELISA measuring IFN-γ. **P < 0.005. (**d**) Cells were separately stained with PE-conjugated anti-IFN-γ Ab and quantitatively analyzed using flow cytometry and CellQuest program.

As our results showed that DCPAB and HPAB suppressed IFN-γ production through regulation of T-bet, we attempted to verify the T-bet-dependency of DCPAB and HPAB functions in IFN-γ suppression. CD4+ T cells were isolated from wild-type (WT) and T-bet knockout (KO) mice and induced to differentiate into Th1 cells in the presence of DCPAB or HPAB. In order to restore T-bet in T-bet-null T cells, we also isolated CD4+ T cells from double transgenic in KO (DTg/KO) mice that restore T-bet expression in a doxycycline-dependent and a T cell-dependent manner^23^ and treated the cells with doxycycline (Fig. 5a). T-bet expression in WT, not KO, Th1 cells was decreased by treatment with DCPAB and HPAB (Fig. 5b). In addition, restored T-bet expression in DTg/KO cells that were treated with doxycycline was decreased by DCPAB or HPAB treatment (Fig. 5b). IFN-γ production was accordingly inhibited in the presence of DCPAB and HPAB in WT Th1 cells, as evidenced by ELISA and intracellular staining (Fig. 5c,d). Although IFN-γ production was dramatically declined in T-bet deficient cells, a fair amount of IFN-γ was still produced in a T-bet-independent way but was not suppressed by treatment with DCPAB and HPAB (Fig. 5c,d), indicating a T-bet-dependent inhibitory functions of DCPAB and HPAB. Furthermore, IFN-γ production was induced by the restoration of T-bet expression in T-bet KO background and the robust production of IFN-γ was subsequently decreased by DCPAB and HPAB (Fig. 5c,d).

### DCPAB and HPAB attenuate T cell-induced inflammatory colitis *in vivo*

**Figure 6:**
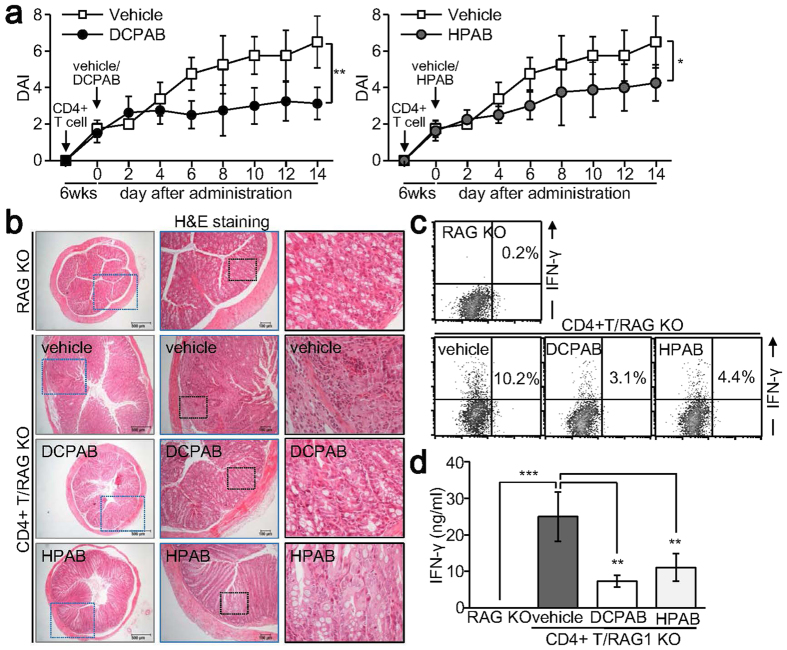
Attenuation of experimental colitis by DCPAB. Naive CD4+ T cells were isolated from WT mice and adoptively transferred into RAG KO mice by intraperitoneal injection. After 6 weeks, CD4+ T/RAG KO mice were daily administered with DCPAB (5 mg/kg) or HPAB (5 mg/kg) for 2 weeks through an intraperitoneal injection. (**a**) Disease activity index was determined in CD4+ T/RAG KO mice treated with vehicle, DCPAB, or HPAB and is expressed as mean ± SD of 8 mice. *P < 0.05, **p < 0.005. (**b**) Colon tissues were sectioned and visualized using H&E staining. Representative images are shown. (**c,d**) Spleen cells were stimulated with anti-CD3 (1 μg/ml) for 24 h. Cells were stained with anti-IFN-γ Ab followed by flow cytometry analysis (**c**). Cell supernatants were collected and used for measuring IFN-γ (**d**). **P < 0.005, ***p < 0.0005.

As DCPAB and HPAB suppressed inflammatory IFN-γ production and Th1 cell development *in vitro*, we evaluated the anti-inflammatory activity of DCPAB and HPAB *in vivo*. Inflammatory colitis was established in immune-deficient recombinase activating gene (RAG) KO mice by adoptively transferring naive CD4+ T cells^24^. Although the disease activity index of colitis was increased in CD4+ T/RAG KO mice, the administration of DCPAB and HPAB attenuated colitis development (Fig. 6a). Daily administration of DCPAB and HPAB significantly suppressed histopathological phenotypes in the inflamed colon (Fig. 6b). IFN-γ production was increased in the spleen of CD4+ T/RAG KO mice but was significantly decreased after the administration of DCPAB and HPAB (Fig. 6c,d). We found that DCPAB and HPAB ameliorated T cell-induced inflammatory colitis *in vivo* through suppression of IFN-γ production.

## Discussion

Our results demonstrated that the novel benzoxazole derivatives, DCPAB and HPAB suppressed the development of Th1 cells by inhibiting T-bet-induced IFN-γ expression, thereby ameliorating inflammatory colitis induced by inflammatory Th1 cells *in vivo*.

IFN-γ, a typical pro-inflammatory cytokine, is predominantly produced by activated T cells. A robust production of IFN-γ was observed in chronic inflammatory disease conditions and its deficiency ameliorated the development of inflammatory colitis^4^,^5^,^6^,^7^, indicating its indispensable role in the initiation and aggravation of inflammatory colitis. IFN-γ expression is mainly regulated at transcriptional level, and T-bet is known to be a master transcription factor for IFN-γ gene transcription. T-bet deficiency impairs IFN-γ production in developing Th cells and prevents their differentiation into mature Th1 cells and attenuates autoimmune responses^22^. The novel benzoxazole derivatives, DCPAB and HPAB, were found to suppress IFN-γ production through inhibition of T-bet expression. DCPAB and HPAB decreased the protein level of T-bet but did not affect T-bet mRNA level, indicating the post-transcriptional or translational regulation of T-bet by DCPAB and HPAB. Indeed, multiple post-translational modification of T-bet including phosphorylation and ubiquitination resulted in differential regulation of Th cell development. T-bet suppression induced by DCPAB was inhibited by MG132 treatment, suggesting the ubiquitination-proteasomal degradation of T-bet protein by DCPAB. It is yet to be clarified that whether DCPAB and HPAB controlled the phosphorylation of T-bet protein or the mRNA stability of T-bet^25^,^26^,^27^. Interestingly, DCPAB, but not HPAB, suppressed the transcriptional activity of T-bet on the IFN-γ gene promoter. It is possible that DCPAB may inhibit the DNA-binding activity of T-bet or transcription complex formation. It also remains to be studied whether and how DCPAB controlled the DNA-binding activity of T-bet and how the structural difference between DCPAB and HPAB affected the transcriptional activity of T-bet. Due to such differences in T-bet regulation, DCPAB more potently suppressed IFN-γ production than HPAB did.

Structurally, DCPAB and HPAB contain the same phenyl amino benzoxazolol, but have different groups in the phenyl ring (two chloride groups and one hydroxyl group, respectively). Because the structures of DCPAB and HPAB are related to their function, the dichlorophenyl ring of DCPAB may be critical for the control of T-bet stability. Although T-bet has been extensively studied and determined to play multiple roles in T cell development, inflammation, and tumor immunity, the structure of T-bet protein has not yet been fully characterized. Structural identification of T-bet protein modifications and DNA-T-bet binding complex will help to identify the functional mechanisms of DCPAB and HPAB underlying IFN-γ suppression in a T-bet-dependent manner. In the future, the structural correlation between the dichlorophenyl group of DCPAB and T-bet binding to the IFN-γ gene promoter should be verified.

CD4+ T cells differentiate into not only Th1 cells, but also into Th2 and Th17 cells upon antigenic stimulation^28^. Th2 and Th17 cells produce IL-4 and IL-17 and control allergic and autoimmune responses, respectively^28^,^29^. Dysregulated and imbalanced generation of Th2 and Th17 cells and overproduction of their signature cytokines cause development of chronic inflammatory and autoimmune diseases *in vivo*^30^. We observed that DCPAB and HPAB specifically suppressed Th1, but not Th2 or Th17, cells. Selective suppression of Th1 cells by DCPAB and HPAB may have beneficial effects on the control of the Th1-mediated inflammatory response, including on inflammatory colitis. However, in addition to IFN-γ, many other cytokines, such as TNF-α, IL-17, IL-6, and IL-23, affect inflammatory colitis development^6^,^31^,^32^,^33^,^34^. Daily injection of DCPAB and HPAB into a T cell-induced colitis model significantly ameliorated disease severity. These *in vivo* results suggested that DCPAB and HPAB may function as potent immune suppressors by suppressing inflammatory responses coordinated by innate and adaptive immune cells. We therefore concluded that DCPAB and HPAB may have therapeutic and preventative benefits in the control of inflammatory diseases. To further evaluate the use of benzoxazole derivatives as anti-inflammatory agents, the broad range effects of DCPAB and HPAB on the differentiation of all subsets of T cells, including regulatory T cells, and on chronic inflammatory and autoimmune diseases should be examined.

## Materials and Methods

### Reagents

All cytokines were purchased from BD Pharmingen (San Diego, CA). Abs against cytokines, T-bet, and β-actin were obtained from BD Pharmingen, Santa Cruz Biotechnology Inc. (Santa Cruz, CA), and Sigma-Aldrich (St Louis, MO).

### Chemicals

DCPAB (2-((3,4-dichlorophenyl)amino)benzo[*d*]oxazol-5-ol) and HPAB (2-((4-hydroxyphenyl)amino)benzo[*d*]oxazol-5-ol) were synthesized by reacting 2-aminophenol with either dichlorophenyl isothiocyanates or ethoxyphenyl isothiocyanates as previously reported^16^. Structures of DCPAB and HPAB were verified using NMR and MS analysis: DCPAB, white powder (31%), mp >250 °C, ^1^H-NMR (DMSO-*d*_6_, 400 MHz) δ 10.855 (s, 1 H), 9.263 (s, 1 H), 8.095 (d, *J* = 2.4 Hz, 1 H), 7.589~7.650 (m, 2 H), 7.269 (d, *J* = 8.8 Hz, 1 H), 6.846 (d, *J* = 2.4 Hz, 1 H), 6.553 (dd, *J* = 2.4 Hz, *J* = 8.4 Hz, 1 H), HR-FABMS Calcd for C_13_H_9_C_l2_N_2_O_2_(M^+^+H): 295.0036, Found: 295.0036; and HPAB, pale gray powder (37%), mp 197~201 °C, ^1^H-NMR (DMSO-*d*_6_, 400 MHz) δ 10.100 (s, 1 H), 9.157 (s, 2 H), 7.459~7.498 (m, 2 H), 7.173 (d, *J* = 8.8 Hz, 1 H), 6.729~6.770 (m, 3 H), 6.458 (dd, *J* = 2.0 Hz, *J* = 8.6 Hz, 1 H), HR-FABMS Calcd for C_13_H_11_N_2_O_3_ (M^+^+H): 243.0765, Found: 243.0764. DCPAB and HPAB were purified with 95% purity (endotoxin-free) and dissolved in 100% DMSO. DCPAB and HPAB was freshly diluted by a factor of 1,000 for treating cells and 0.1% DMSO was used as the control (vehicle).

### Mice

Wild-type (WT) C57BL6 mice (The Jackson Laboratories, Bar Harbor, MN), T-bet knockout (KO)^24^, and Double transgenic knockout (DTg/KO)^26^ were housed and bred at Ewha Womans University under specific pathogen free conditions and sacrificed before the experiments in compliance with the Institutional Animal Care and Used Committee (IACUC) guidelines at Ewha Womans University (2012-01-071, 2013-01-013, and 2014-01-011). Recombination activating gene (RAG) KO mice (The Jackson Laboratories) were also housed in an animal facility at Ewha Womans University and were used for adoptive transfer with naïve CD4+ T cells according to the protocol approved by IACUC (2012-01-072).

### *In vitro* culture of spleen cells and differentiation of CD4+ T cells into Th1 cells

Single-cell suspensions were obtained from spleen of WT (male, 6 to 8 weeks of age) mice and cultured in RPMI 1640 media (HyClone Laboratories, Logan, UT). Cells (5 × 10^6^ cells/well for 6-well plate) were stimulated with anti-CD3 Ab (1 μg/mL, BD Pharmingen) in the presence of vehicle (0.1% DMSO), DCPAB (10 μM), or HPAB (10 μM) for 24 h. CD4+ T cells were isolated from single-cell suspensions of lymph node and spleen using miniMACS CD4 microbeads (Miltenyi Biotec Inc., San Diego, CA). Cells were stimulated with plate bound anti-CD3 (2 μg/ml) and anti-CD28 (1 μg/ml) for 24 h and additionally treated with recombinant human IL-2 (10 U/mL), IL-12 (2 ng/mL), and anti-IL-4 Ab (5 μg/ml) for Th1 differentiation. Instead of IL-12 and anti-IL-4 Ab, cells were treated with IL-4 and anti-IFN-γ Ab for Th2 cell differentiation, or skewed into Th17 cells by treatment with TGF-β and IL-6. Cells were additionally incubated with DCPAB or HPAB for an additional 4–5 days and were stimulated with anti-CD3 (1 μg/ml) for 24 h. Cell supernatants were analyzed by ELISA and cells were used for intracellular cytokine staining analysis or total RNA preparation.

### Cytotoxicity assay

Spleen cells were stimulated with anti-CD3 and incubated with either DCPAB or HPAB for 24 h. Cells were additionally treated with EZ-Cytox reagent (EZ-CYTOX cell viability assay kit, Daeil lab, Seoul, Korea) for 2 h. Cell supernatants were harvested and measured using microplate reader at 450 nm according to the manufacturer’s instructions. Cell viability was calculated and expressed as percentage after normalization with vehicle-treated control.

### Cytokine measurement by ELISA

An ELISA plate was pre-coated with purified anti-IFN-γ Ab (1 μg/ml) and incubated with cell supernatants for 1 h. The plate was washed and loaded with biotinylated anti-IFN-γ (1 μg/ml) Ab, and subsequently incubated with streptavidin-conjugated alkaline phosphatase (1 μg/ml). The substrate for phosphatase was added and color development was detected at 405 nm using a microplate reader (Molecular Devices, Sunnyvale, CA). The known amount of standard cytokine was used to construct a standard curve.

### Intracellular cytokine staining

Differentiated Th1 cells were pre-treated with monensin (4 μM) for 3 h and collected, followed by fix in 4% paraformaldehyde. Cells were treated with permeablization buffer (0.1% saponin, 0.1% sodium azide, and 1% fetal bovine serum in phosphate buffered saline) and incubated with phycoerythrin (PE)-conjugated anti-IFN-γ Ab (2 μg/ml) for 30 min on ice in dark. After washing, the cells were analyzed by flow cytometry and CellQuest software (BD Pharmingen).

### Reverse transcription and real-time PCR analysis

Total RNA was isolated from the cells using TRIzol reagent (Invitrogen, San Diego, CA) and reversely transcribed into cDNA (Promega, Madison, WI). Quantitative real time-PCR was performed with a SYBR Green qPCR Mix (TOYOBO, Japan) and detected by an ABI PRISM 7300 sequence detection system (Applied Biosystems, Carlsbad, CA). Primers were used as follows: IFN-γ, 5′-agcaacagcaaggcgaaaa-3′ and 5′-ctggacctgtgggttgttga-3′; IL-4, 5′-acaggagaagggacgccat-3′ and 5′-gaagccctacagacgagctca-3′; IL-17, 5′-ctggaggataacactgtgagagt-3′ and 5′-tgctgaatggcgacggagttc-3′; T-bet, 5′-gccagggaaccgcttatatg-3′ and 5′-gacgatcatctgggtcacattgt-3′; and β-actin, 5′-agagggaaatcgtgcgtgac-3′ and 5′-caatagtgatgacctggccgt-3′. Relative transcript levels of the specific genes were calculated by transforming Ct values from real time-PCR to quantities using the comparative Ct method and dividing the quantities by the actin values for normalization. The relative expression level was presented as a ratio relative to vehicle control.

### Reporter gene assay

Highly transfectable human embryonic kidney (HEK) 293 T cells were transfected with an IFN-γ promoter-linked reporter gene (pIFN-γ-luc) and T-bet expression vector. The pCMVβ reporter gene that expresses β-galactosidase activity was also transfected for normalization of transfection efficiency. Luciferase and β-galactosidase activity were measured using a luciferase assay kit (Promega) and a Galacto-Light (Tropix), respectively. Relative luciferase activity was calculated after normalization with β-galactosidase. Mock transfection was set to 1 by fold induction.

### Development of T-cell induced colitis model and histological examination

Naive CD4+ T cells (CD4^+^ CD62L^+^ CD44^−^) were isolated from the lymph node of C57BL6 mice (male, 6 weeks of age, Jackson Laboratories) using naïve CD4+ T cell enrichment column (R&D systems, Minneapolis, MN) and the cells (0.5 × 10^6^ cells/mouse) were then adoptively transferred into RAG KO mice (male, 6 weeks of age, n = 24)^24^ via intraperitoneal injection. After 6 weeks, CD4+ T cell-transferred RAG KO mice (CD4+ T/RAG KO, n = 8) were administered with vehicle (50% DMSO), DCPAB (5 mg/kg), or HPAB (5 mg/kg) via daily intraperitoneal injection and examined for 2 weeks. Disease activity index was determined by combination of score of weight loss (no loss, 0; 1–5%, 1; 5–10%, 2; and 10–20%, 3), stool consistency (normal, 0; loose, 2; and diarrhea, 4), bleeding (no blood, 0; brown, 1; reddish, 2; and gross bleeding, 3), and histological damage (no epithelial loss, 0; crypt destruction 2; and mucosal infiltration of immune cells, 4). Colonic tissues were harvested, sectioned, and stained with hematoxylin and eosin (H&E) staining solution, followed by microscopic observation^35^.

### Statistical analysis

The results were given as mean ± SD from three or four independent experiments. The statistical significance of results was determined using a one-way ANOVA or two-tailed unpaired Student’s t-test. P value less than 0.05 (P < 0.05) was considered statistically significant.

## Conclusion

The novel benzoxazole derivatives, DCPAB and HPAB, share a common amino phenyl benzoxazole structure and specifically suppressed IFN-γ production and Th1 cell differentiation through the inhibition of T-bet expression. DCPAB additionally inhibited T-bet activity and thus more potently suppressed IFN-γ production than HPAB did. DCPAB and HPAB attenuated inflammatory colitis induced by T cells *in vivo*, indicating the therapeutic effects of DCPAB and HPAB on chronic inflammatory diseases.

## Additional Information

**How to cite this article**: Oh, Y. J. *et al*. Novel benzoxazole derivatives DCPAB and HPAB attenuate Th1 cell-mediated inflammation through T-bet suppression. *Sci. Rep.* **7**, 42144; doi: 10.1038/srep42144 (2017).

**Publisher's note:** Springer Nature remains neutral with regard to jurisdictional claims in published maps and institutional affiliations.

## Supplementary Material

Supplementary Information
