# Supplementary material for: Novel benzoxazole derivatives DCPAB and HPAB attenuate Th1 cell-mediated inflammation through T-bet suppression
Source: Sci Rep. 2017 Feb 7;7:42144. doi: 10.1038/srep42144 (PMC5294415; doi:10.1038/srep42144)
Supplement: Supplementary Information [file srep42144-s1.pdf]

**Novel benzoxazole derivatives DCPAB and HPAB attenuate Th1 cell-mediated inflammation through T-bet suppression**

Yeon Ji Oh[1], Darong Kim[1], Sera Oh[1], Eun Jung Jang[1], Hee Yeon Won[1], Hana Jeong[1], Mi Gyeong Jeong[1], Hea-Young Park Choo[1], and Eun Sook Hwang[1*]

[1]College of Pharmacy and Graduate School of Pharmaceutical Sciences, Ewha Womans University, Seoul 03760, Korea

Figure 4a

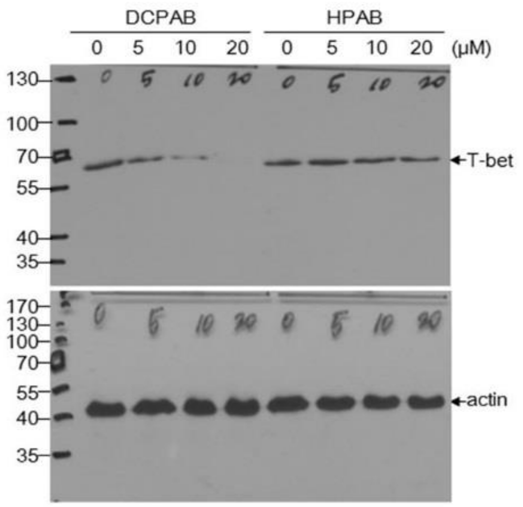


Figure 4c

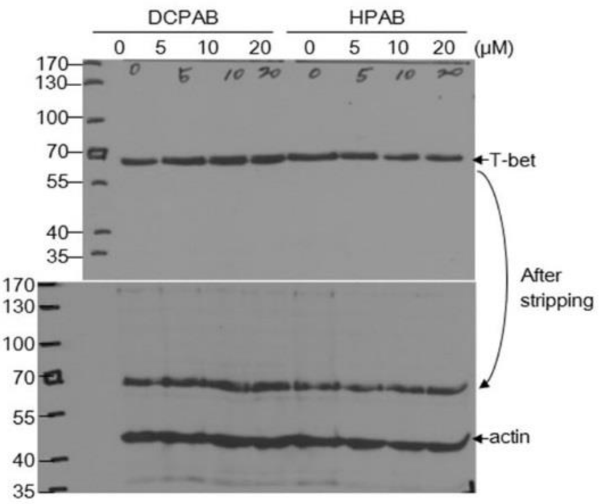


**Supplementary Figure 1.** Uncropped original images of immunoblot analysis as shown in Figure 4a and 4c.

Figure 5b

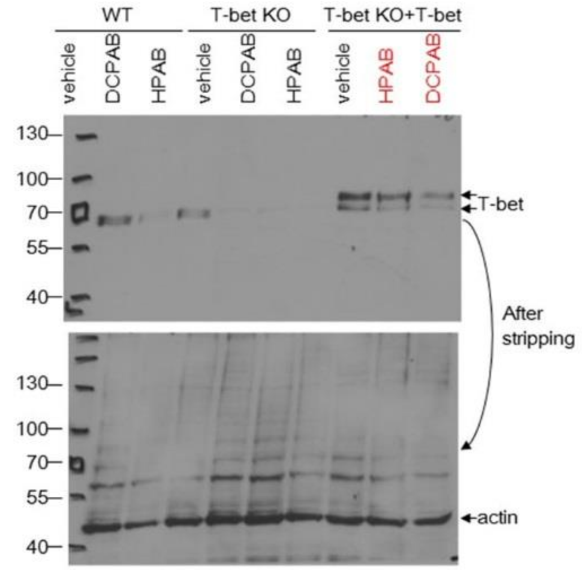


**Supplementary Figure 2.** Uncropped images of immunoblot analysis as shown in Figure 5b.

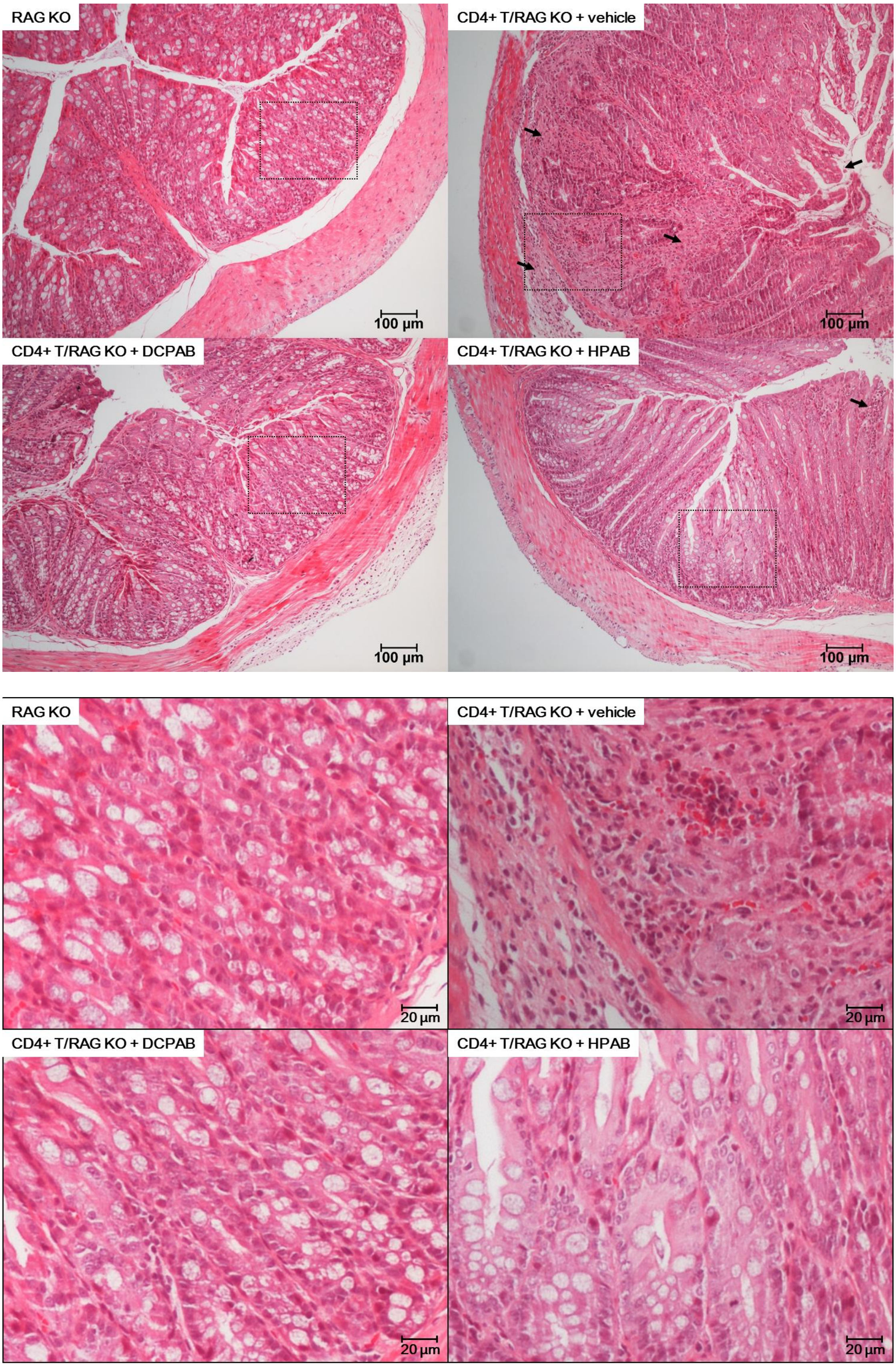


**Supplementary Figure 3.** Unabridged microscopic images of colonic tissues as shown in Figure 6b.
